# Analysis of RNA splicing defects in PITX2 mutants supports a gene dosage model of Axenfeld-Rieger syndrome

**DOI:** 10.1186/1471-2350-7-59

**Published:** 2006-07-11

**Authors:** Nicole L Maciolek, Wallace LM Alward, Jeffrey C Murray, Elena V Semina, Mark T McNally

**Affiliations:** 1 Department of Microbiology and Molecular Genetics, Medical College of Wisconsin, 8701 Watertown Plank Road, Milwaukee, WI 53226, USA; 2 Department of Pediatrics, Medical College of Wisconsin, 8701 Watertown Plank Road, Milwaukee, WI 53226, USA; 3 Department of Ophthalmology, University of Iowa, Iowa City, IA 52242, USA; 4 Department of Pediatrics, University of Iowa, Iowa City, IA 52242, USA

## Abstract

**Background:**

Axenfeld-Rieger syndrome (ARS) is associated with mutations in the *PITX2 *gene that encodes a homeobox transcription factor. Several intronic *PITX2 *mutations have been reported in Axenfeld-Rieger patients but their effects on gene expression have not been tested.

**Methods:**

We present two new families with recurrent *PITX2 *intronic mutations and use *PITX2c *minigenes and transfected cells to address the hypothesis that intronic mutations effect RNA splicing. Three *PITX2 *mutations have been analyzed: a G>T mutation within the AG 3' splice site (ss) junction associated with exon 4 (IVS4-1G>T), a G>C mutation at position +5 of the 5' (ss) of exon 4 (IVS4+5G>C), and a previously reported A>G substitution at position -11 of 3'ss of exon 5 (IVS5-11A>G).

**Results:**

Mutation IVS4+5G>C showed 71% retention of the intron between exons 4 and 5, and poorly expressed protein. Wild-type protein levels were proportionally expressed from correctly spliced mRNA. The G>T mutation within the exon 4 AG 3'ss junction shifted splicing exclusively to a new AG and resulted in a severely truncated, poorly expressed protein. Finally, the A>G substitution at position -11 of the 3'ss of exon 5 shifted splicing exclusively to a newly created upstream AG and resulted in generation of a protein with a truncated homeodomain.

**Conclusion:**

This is the first direct evidence to support aberrant RNA splicing as the mechanism underlying the disorder in some patients and suggests that the magnitude of the splicing defect may contribute to the variability of ARS phenotypes, in support of a gene dosage model of Axenfeld-Rieger syndrome.

## Background

Axenfeld-Rieger syndrome (ARS) is an autosomal-dominant disorder with complete penetrance but variable expressivity, and is one of the developmental conditions of Axenfeld-Rieger spectrum. The spectrum is defined on the basis of specific eye anomalies that include prominent annular white line near the limbus at the level of Descemet membrane (posterior embryotoxon), hypoplastic iris, irido-corneal adhesions and glaucoma [1-5]. Diagnosis of ARS is established when the above-described ocular features are accompanied by other systemic abnormalities, most commonly craniofacial, dental and umbilical defects. Craniofacial anomalies usually consist of maxillary hypoplasia, thin lip and dysplastic ears. Dental defects vary from small teeth to complete anodontia with missing lateral mandibular incisors being the most common feature. Umbilical anomalies may range from isolated redundant skin at the site of the umbilicus to severe hernias or omphalocele. Among other associated anomalies, pituitary and cardiac defects, hearing loss, hydrocephalus and hypospadius have been reported [6-8].

Axenfeld-Rieger spectrum is a heterogeneous condition. Mutations in *PITX2 *(4q25), *FOXC1 *(6p25), *PAX6 *(11p12), and a yet to be identified gene at 13q14 have been shown to result in Axenfeld-Rieger isolated eye anomalies as well as the complete syndrome [9-15]. Ocular manifestations of *PITX2 *mutations show broad variability, both between and within families. To date, the reported phenotypes include Rieger and Axenfeld anomaly, iris hypoplasia, iridogoniodysgenesis, Peters' anomaly, aniridia and ring dermoid of cornea [9,16-27]. The *PITX2 *gene encodes a homeodomain-containing transcription factor and spans about 20 kb of genomic sequence and includes six exons that encode four alternative transcripts that arise by alternative splicing and the differential use of three promoters [28]. Although gain-of-function mutations have been reported [19,29], a deficiency in normal PITX2 protein (haploinsufficiency) is suggested to be the major mechanism of ARS. This is supported by the presence of large deletions that include *PITX2 *in some Axenfeld-Rieger patients and functional studies of proteins derived from mutant alleles. A correlation between the dosage of normal PITX2 protein and the severity of the phenotype was noted [19,20,30,31]. Most of the human *PITX2 *mutations described thus far affect regions encoding the homeodomain- or C-terminal domains, although a few intronic mutations have been reported (see references above).

Pre-mRNA splicing is the process whereby introns are removed and exons are joined to produce mature mRNA. RNA splicing is facilitated by a large macromolecular machine, the spliceosome, which recognizes conserved sequences at intron/exon borders, including the 5' and 3' splice sites and branchpoint sequence [32]. In a few instances, *PITX2 *mutations have been identified in introns either at or in close proximity to splice sites (ss) associated with the last two exons. These observations suggest that splicing defects might explain the syndrome in these individuals. To date, only coding region mutations in *PITX2 *have been investigated. Here, we report the identification of two new human families with intronic *PITX2 *mutations and present an analysis of the effects of intronic mutations on *PITX2 *mRNA splicing. The data suggest that aberrant RNA splicing underlies the disorder in six families and that the degree of aberrant splicing may contribute to the variability of Axenfeld-Rieger syndrome phenotypes.

## Methods

### Identification of PITX2 mutations

DNA samples obtained from new patients with ARS and anomaly were screened for PITX2 gene mutations in exons and at least 100-bp into adjacent intron regions as previously described [9]. DNA was isolated from blood spots using the QIAGEN QIamp^® ^DNA mini kit following the dried blood spot protocol. PCR was conducted in a GeneAmp^® ^PCR system 9700 in 30 μl reactions containing 1.5 mmol/l Mg 2+, 40–100 ng DNA, Biolase Reaction Buffer (Bioline), 0.25 mM each dNTP, 1.5 units Biolase DNA polymerase (Bioline) and 0.2 μM of each oligodeoxynucleotide primer. Cycling profile was one cycle of 94°C for five minutes, 30 cycles of 94°C for 45 seconds, 56°C for 45 seconds, and 72°C for 45 seconds, and one cycle of 72°C for ten minutes. Products were either purified using Millipore MultiScreen^® ^Separations System or sequenced directly. Products were sequenced bidirectionally with an ABI Prism^® ^3700 DNA sequencer using ABI Prism^® ^BigDye™ Terminator Cycle Sequencing Ready Reaction Kits. The 20 μl reactions contained 5% DMSO, 1 μl of PCR product DNA, and 0.32 μM oligodeoxynucleotide primer. Cycling profile was conducted in a GeneAmp^® ^PCR System 9700. Cycling profile was one cycle of 96°C for 30 seconds, 35 cycles of 96°C for ten seconds, 50°C for 5 seconds and 60°C for four minutes. Extension products were purified using Agencourt CleanSEQ^® ^Reaction Clean up. PCR products were then sent to Agencourt, Inc to be sequenced. Sequences were manually examined for mutations and confirmed by additional independent amplification and sequencing.

### PITX2 DNA constructs

A primer positioned at g.16944 of the *PITX2 *genomic sequence (GenBank accession number AF238048) and a downstream primer at g.17393 were used to PCR-amplify a 473 bp fragment that included part of exon 1b and the downstream intron using human genomic DNA as a PCR template; EcoRI and BamHI sites were appended to the 5' and 3' ends, respectively. Primers are listed in Table 1. A 653 bp fragment containing exon 4 and surrounding intron was amplified using an upstream primer at position g.17812 (with a 5' BamHI site appended) and a downstream primer at g.18439 containing a 3' KpnI site. A 713 bp fragment containing a portion of exon 5 and the upstream intron was generated using an upstream primer positioned at g.20001 and a downstream primer at g.21147; 5' KpnI and 3' HindIII sites were added to primers. Cycling profile was one cycle of 94°C for 45 s, 30 cycles at 94°C for 45 s, 60°C (55°C for exon 1b) for 45 s, and 72°C for 45 s, and one cycle of 72°C for 10 min. Fragments were inserted individually into pGEM-3Z (Promega) using the same restriction sites, and plasmids were sequenced using an ABI PRISM BigDye Terminator v3.1 cycle sequencing kit (Applied Biosystems). Using the appended restriction sites, exon/intron fragments were excised from pGEM-3Z and shuttled sequentially into the same sites of pcDNA3.1/myc-His(-)A (Invitrogen) to create the PITX2c minigene (Figure 2B). Mutant minigenes were constructed by overlap PCR using the above pGEM-3Z constructs as template [33]. Sense and antisense primers containing the desired mutations were used with the appropriate outside primer for PCR to synthesize half-substrates. Mutant exon fragments were inserted into pGEM-3Z and mutations were verified by DNA sequencing. The mutant fragments were then used to replace the WT fragment in the minigene construct as described above. N-terminal FLAG-epitope tagged minigenes were constructed by excising the entire minigene from the pcDNA constructs using SalI and a partial EcoRI digestion, and shuttling the fragments into the same sites of p3X-FLAG CMV 7.1 (Sigma).

**Table 1:** Oligonucleotides used in this study

**Name**	Sequence*	**Purpose**
Ex-1b-f	GCGAATTCCAGTAGCCAAGGACTAGTAG	Forward and reverse primers to make exon 1b fragment
Int-1b-r	GCGGATCCAGAATTGCTCGCGCCCTTAG	
Int-1b-f	GCGGATCCAGTGAATGTGCCGCTGCAGT	Forward and reverse primers to make exon 4 fragment
Int-4-r	GCGGTACCTCGGAGAGGGAACTGTAATC	
Int-4-s	GCGGTACCTGGCTGAGTGATCAAACCGT	Forward and reverse primers to make exon 5 fragment
Ex-5-r	CGAAGCTTGGCGGCGCGTAAGGACAGG	
IVS4+5G>C-f	GAGTCCGGGTAGcAGCCAGCACGGAG	Forward and reverse overlap PCR primers to make IVS4+5G>C mutant
IVS4+5G>C-r	CTCCGTGCTGGCTgCTACCCGGACTC	
IVS5-11A>G-f	CTCCCTTGCCCCAgCCGCCCCCAGG	Forward and reverse overlap PCR primers to make IVS5-11A>G mutant
IVS5-11A>G-f	CCTGGGGGCGGcTGGGGCAAGGGAG	
IVS4-1G>T-f	CGTTTTCAtAGAAAGAT	Forward and reverse overlap PCR primers to make IVS4-1G>T mutant
IVS4-1G>T-f	ATCTTTCTaTGAAAACG	
T7 promoter	TAATACGACTCACTATAGGG	Forward primer to pcDNA T7 promoter to detect minigene mRNA
PCREx-1b-f	TGTCGGCCGTCTCCTCATCTTCC	Forward and reverse primers to detect endogenous and minigene mRNA
PCREx-5-r	TTGCGCTCCCTCTTTCTCCATTTG	
GFP-f	GACGGCAACATCCTGGGGCACAAG	Forward and reverse primers to GFP
GFP-r	CGGCGGCGGTCACGAACTCC	
GAPDH-f	TGATGACATCAAGAAGGTGGTGAAG	Forward and reverse primers to GAPDH
GAPDH-r	TCCTTGGAGGCCATGTGGGCCAT	

*Primers are 5' to 3', mutations are in lowercase

**Figure 1:**
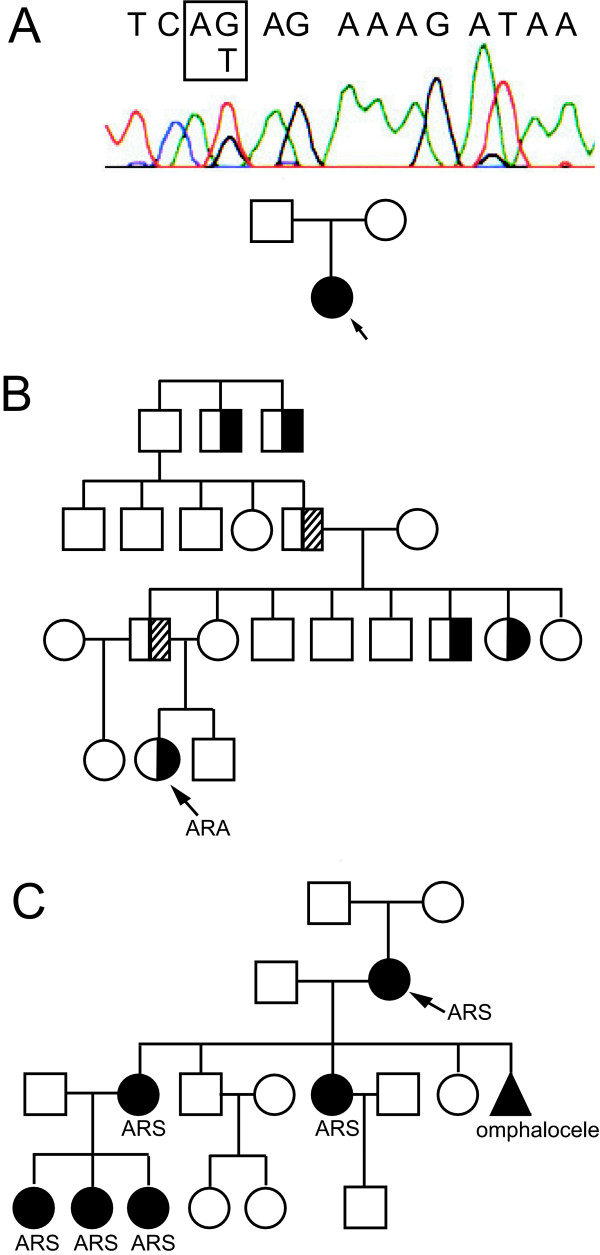
**Axenfeld-Rieger pedigrees with intronic mutations in *PITX2***. A. Pedigree of family 0175 and sequence of genomic DNA of the patient showing an intronic mutation in the AG dinucleotide at the 3' ss splice junction of exon 4 (boxed). Proband is indicated with an arrow. B. Pedigree of family 689. Proband is indicated with an arrow. Individuals affected with an isolated ocular phenotype according to family history are indicated by half-shaded circles or boxes. Individuals affected with subclinical phenotypes who are likely mutation carriers are indicated with half-striped boxes. ARA, Axenfeld-Rieger anomaly. C. Pedigree of family 2 with clinical features. Proband is indicated with an arrow. Individuals affected with classic Axenfeld-Rieger syndrome (includes the triad of ocular, dental and umbilical anomalies) are indicated as shaded circles or boxes. ARS, Axenfeld-Rieger syndrome. The shaded triangle indicates a miscarriage of a child of unknown sex.

**Figure 2:**
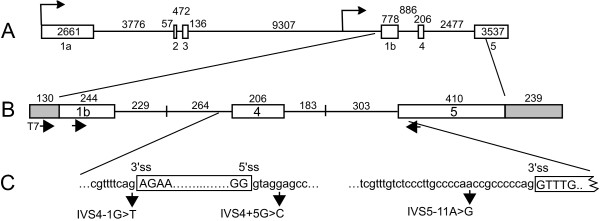
***PITX2 *gene and minigene structure**. A. Schematic of *PITX2 *gene organization (drawn to scale). Open boxes represent exons, lines represent introns. The sizes of exons and introns are shown. The *PITX2 *gene has two major promoters (arrows) and six exons. Alternative splicing and transcription start sites give three major isoforms (not shown). *PITX2c *uses the downstream promoter and produces an mRNA containing exon 1b, 4, and 5. B. Schematic of *PITX2c *minigene. The minigene contains a portion of exon 1b, exon 4, part of exon 5, and uses the CMV promoter and bGH polyadenylation site from the pcDNA3.1/*myc*-His(-)A vector (transcribed vector sequences are represented by shaded boxes). The lengths of intron regions associated with each exon are shown with vertical lines indicating the fusion of two fragments. The positions of the T7 primer to vector sequences, and primers to PITX2 exons 1b and 5 that were used for RT-PCR are shown below the diagram. C. Expansion of (B) showing the names and locations of three different intronic point mutations identified in ARS patients. The sequence surrounding the 5' and 3' splice sites of exon 4, and the 3' ss of exon 5 is shown. Mutations in patients 0175 (IVS4-1G>T), 689 or 2 (IVS4+5G>C), and 4 (IVS5-11A>G) are indicated by arrows and the nucleotide changes are shown.

### Transfection and RNA analysis

HEK293 and HeLa cells were grown in minimal essential growth medium supplemented with 10% fetal calf serum. Human cornea stromal cells were grown in Dulbecco's modified Eagle medium supplemented with 10% fetal calf serum. Cells were grown to about 60% confluence in 6 cm dishes (HEK293 and HeLa) or 6 well plates (cornea stromal cells) and transfected with 2 μg of minigene DNA and 0.5 μg GFP using the calcium phosphate method (Amersham Biosciences) for HEK293 and HeLa cells, or Lipofectamine (Invitrogen) for cornea stromal cells. Total RNA was harvested 48 h later using the Qiagen RNeasy kit. For RT-PCR, reverse transcription was performed on 1 μg of total RNA using oligo dT in a volume of 25 μl. For PCR, 2.5 μl of the reverse transcription mixture was used in a 25 μl PCR reaction using the T7 primer to pcDNA3.1/myc-His(-)A vector and to exon 5 (for minigene mRNA) or to exon 1b and exon 5 (for endogenous plus minigene mRNA), and primers to GFP or cellular GAPDH. Cycling conditions were one cycle of 94°C for 45 seconds, 30 cycles of 94°C for 45 seconds, 60°C for 45 seconds, and 72°C for 45 seconds, and one cycle of 72°C for 45 seconds. To evaluate the accuracy of splicing of WT and mutant mRNA, RT-PCR products were cloned and sequenced. RT-PCR products were blunted with Klenow fragment and then digested with EcoRI, which cuts at a site located upstream of exon 1b. The products were inserted into the EcoRI and SmaI sites of pGEM-3Z and individual clones were sequenced. To assess minigene splicing by RNase protection assay, a 700 bp riboprobe was made by in vitro transcription using T7 polymerase and HindIII-linearized pGEM-3Z containing the exon/intron 4 fragment. This includes 264 nt and 183 nts of upstream and downstream intron, respectively, that flank the 206 nt exon 4. A 371 nt GFP riboprobe was made using SP6 polymerase and EcoRI-linearized pGEM-3Z containing a GFP fragment. RNase protection assays were carried out as described previously [34] using 2.5 μg RNA. Correct splicing generates a ~206 nt protected band and intron retention yields a ~386 nt band. The GFP probe generates a ~309 nt protected band. Results were visualized by autoradiography and quantitated with a Storm 820 PhosphorImager (Amersham Biosciences).

### Western Blotting

HEK 293 cells were transfected with 2 μg p3X-FLAG minigene DNA and 1 μg pEGFP as described. Cells were washed with phosphate buffered saline, harvested by scraping, the cells were resuspended in water, an equal volume of 2× solubilizing buffer (2% SDS, 10% β-mercaptoethanol, 5% glycerol, 50 μM Tris, pH 7, 0.005% bromphenol blue) was added, and samples were sonicated for five seconds. Samples were subjected to 10% SDS-PAGE and transferred to nitrocellulose membrane by electrotransfer. Immunoblotting was done as described [33], blots were blocked with nonfat dry milk, incubated with anti-FLAG M2 monoclonal primary antibody (Sigma) or anti-GFP monoclonal primary antibody (Covance), and after washing, goat anti-mouse IgMμ HRP-conjugate secondary antibody (Upstate) was applied. Blots were developed using Supersignal West Pico Chemiluminescent Substrate (Pierce). Results were visualized by autoradiography or images were obtained using a Fluorchem IS-8800 (Alpha Innotech).

## Results

### Identification of two new families with intronic PITX2 mutations

DNA samples obtained from new patients with Axenfeld-Rieger syndrome and anomaly were screened for mutations in exons and at least 100-bp in the adjacent intron regions. Two new families with intronic *PITX2 *mutations have been identified. The first mutation was identified in a patient (0175) affected with ARS (Figure 1A). The patient displayed all classic features of this condition and some additional defects: Rieger anomaly of the eye, wide-spaced eyes, thin upper lip, hypoplastic maxilla, dental defects, redundant periumbilical skin, and an anteriorly placed anus. The mutation identified in this patient is a G>T change in the -1 position of the 3' ss associated with exon 4 (IVS4-1G>T) (g.18072G>T as seen in *PITX2 *sequence GenBank # AF238048; Figures 1A and 2C). Both parents display a normal phenotype; testing of the patient's mother confirmed normal PITX2 sequence (data not shown) and the father was not available for testing. This mutation was reported previously in an ARS patient with severe ocular malformations, underdeveloped maxilla and redundant umbilical skin [25]. Perveen et al. [18] reported a G>C mutation at the same position and affected individuals from this family demonstrated severe ARS phenotypes: severe iris hypoplasia emulating aniridia in one patient and joint hypermobility and an anteriorly placed anus in another individual were reported.

The second mutation was found in patient 689, who displayed Axenfeld-Rieger anomaly in one eye only and no craniofacial, dental or umbilical abnormalities. The patient had normal ocular pressure and vision, the right eye had a posterior embryotoxon visible on slit lamp exam with iris sheets sweeping up to the embryotoxon inferiorly, and no iris hypoplasia was noted. Slit lamp exam of the left eye demonstrated normal development. Clinical family history of this patient contains records of developmental ocular defects but dental or umbilical anomalies were never noted (Figure 1B). The mutation was identified as a G>C substitution at the +5 position of the 5' ss of *PITX2 *exon 4 (IVS4+5G>C)(g.18283G>C as seen in *PITX2 *sequence GenBank # AF238048; Figures 1B and 2C). The same change was previously reported in a patient affected with the complete ARS [9]. Collection and examination of clinical information from this family indicated a substantial family history of complete ARS (pedigree and clinical diagnoses are shown in Figure 1C).

### Analysis of PITX2c RNA splicing using a minigene construct

Most Rieger syndrome patients have point mutations in the coding region of *PITX2 *[9] and most of these affect the DNA binding homeodomain [35]. Previous work and the data above brings to eight the number of families with mutations that do not affect the protein coding potential of the gene, and the number of different mutations is now five [9,17,18,21,25]. We set out to examine three recurrent intronic mutations, each of which was described in two unrelated families of Axenfeld-Rieger spectrum, and to correlate the phenotypes with potential molecular defects in pre-mRNA splicing. The IVS4-1G>T and IVS4+5G>C mutations were described above. A third mutation studied was an A>G change 11 nt upstream of the 3'ss of exon 5 (IVS5-11A>G) that was originally described in family 4 by Semina et al. [9] and later by Borges et al. [21] who reported the same change in another family (family 5); both families displayed classic features of ARS.

The *PITX2 *gene is located at 4q25 and numerous isoforms are produced through the use of alternative promoters and extensive alternative splicing of the six exons (Figure 2A) [28]. *PITX2 *is expressed developmentally and in some adult tissues but not blood (EVS, unpublished) and thus patient samples to analyze splicing directly are not readily available. To overcome this limitation, a minigene for expression in tissue culture cells was designed that included the exons that comprise one of the common *PITX2 *isoforms, *PITX2c*, (Figure 2B) [36]. Since the signals required for constitutive splicing are usually within ~100 nucleotides of splice junctions [32,37], the introns were truncated to facilitate DNA cloning. Because there is no evidence that the extensive 5' and 3' untranslated regions are needed for constitutive splicing, exons 1b and 5 were also truncated; exon 1b starts 40 nt upstream of the translation start codon and the exon 5 fragment ends with the last amino acid codon. Minigene splicing was analyzed in HeLa and HEK293 cells since they have been used extensively to study constitutive RNA splicing. Additionally, cornea stromal cells (generously provided by Dr. Watsky, University of Tennessee) [38] were used since *PITX2 *is expressed during corneal development and this cell line endogenously expresses *PITX2*, and thus may more faithfully reproduce *PITX2 *splicing that occurs in patients.

**Figure 3:**
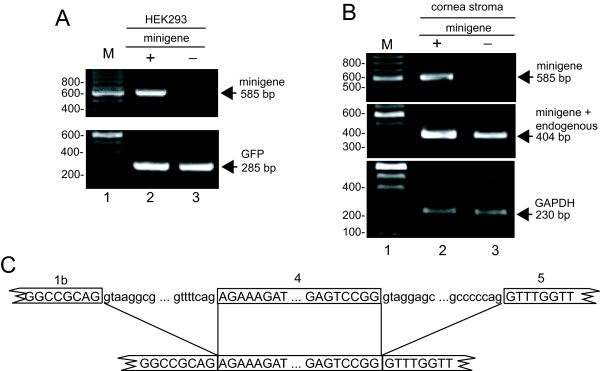
**The *PITX2c *minigene is expressed and the mRNA splices normally**. A. HEK293 cells were transfected with a GFP transfection control plasmid with (+) or without (-) 2 μg of the minigene. Transfected-cell RNA was isolated and subjected to RT-PCR with primers to the minigene (top) or GFP (bottom) and the products were resolved on an agarose gel. The position of correctly spliced product for the minigene and GFP is shown at the right. M, 100 bp size standards with selected sizes shown. B. Human cornea stromal cells were either mock transfected (-) or transfected with 2 μg of minigene DNA (+). RNA was isolated and analyzed by RT-PCR with primers that amplify only minigene mRNA (top) or those that amplify both minigene and endogenous *PITX2c *mRNA (middle), or to cellular GAPDH (bottom), and products were resolved on an agarose gel. Primers are depicted in Figure 2B. M, 100 bp size standards with selected sizes shown. The positions of correctly spliced products are shown at the right. C. Sequence of spliced products. RT-PCR products were cloned and sequenced. At the top is a schematic of the pre-mRNA with exon sequences boxed and in uppercase and intron sequences in lowercase. Lines extend from splice sites to the spliced product sequence shown at the bottom.

HeLa and HEK293 cells were mock transfected or transfected with 2 μg of minigene DNA and/or a GFP-expressing transfection control plasmid and RT-PCR was performed on RNA isolated 40–48 h later. While *PITX2c *is not expressed in these cell types (data not shown), to ensure that only minigene RNA was detected, an upstream primer complementary to vector sequence was used in combination with a primer specific to *PITX2 *exon 5. Correct splicing would result in a 585 bp RT-PCR product whereas unspliced RNA would produce a 1564 bp product. The 585 bp band was observed only from transfected HEK293 cells and analysis of GFP expression demonstrated equivalent transfection efficiency and gel loading (Figure 3A, lanes 2 and 3). Similar results were obtained with HeLa cell RNA (data not shown). These results suggest that the HeLa and HEK293 cells faithfully express correctly spliced minigene RNA. As an additional test to verify the utility of the minigene, human cornea stromal cells were similarly examined. The primer pair specific for the minigene resulted in the 585 bp spliced product only in transfected cells (Figure 3B, lanes 2 and 3, top) whereas a primer pair that does not distinguish between transfected and endogenous *PITX2c *mRNA produced a 404 bp product that was present in both but more abundant in transfected cells, consistent with correct splicing of endogenous and transfected *PITX2 *mRNA in corneal cells (lanes 2 and 3, middle). Analysis of cellular GAPDH showed equal sample loading (Figure 3B, bottom).

To further assess the accuracy of minigene mRNA splicing in HEK293 and human cornea stromal cells, the RT-PCR products were cloned and three and four independent clones were sequenced, respectively. Each sequence showed correct minigene splicing (Figure 3C). These data indicate that the *PITX2c *minigene was efficiently and accurately spliced and therefore validated the system for determining the effects of patient mutations on RNA splicing. This system would not address potential tissue-specific splicing defects that may be important for ARS.

### A G>T mutation at the 3' splice site of exon 4 shifts splicing 2 nt downstream

**Figure 4:**
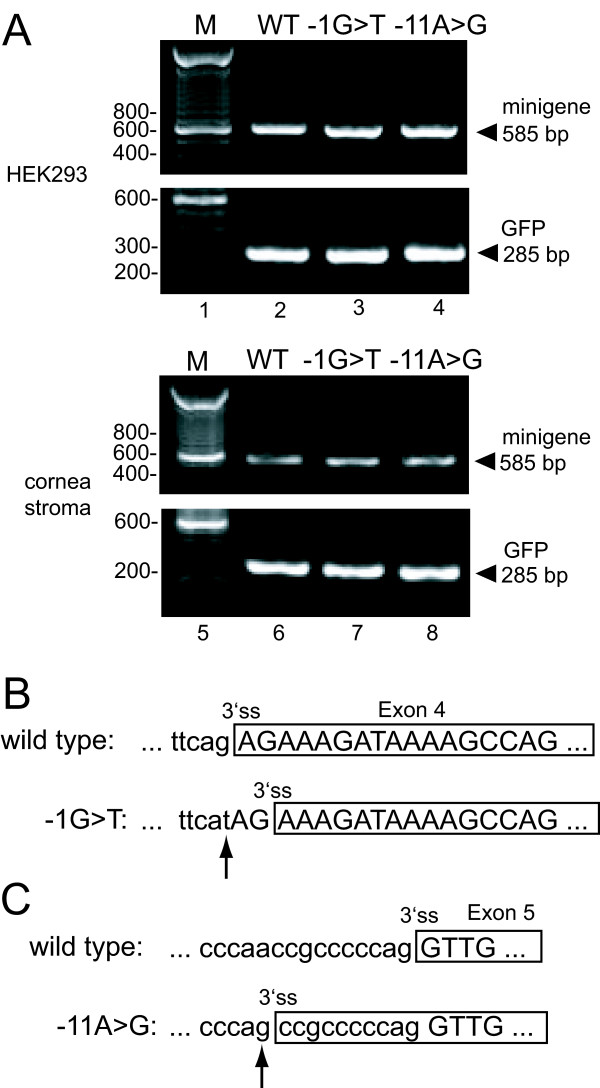
**Mutants IVS4A-1T and IVS5-11A>G splice aberrantly**. A. Analysis of transfected-cell RNA by RT-PCR. RNA from HEK293 cells (lanes 2–4) or human cornea stromal cells (lanes 6–8) transfected with GFP and the indicated minigene was analyzed by RT-PCR and products were resolved on an agarose gel. The position of correctly spliced minigene product (585 bp) and GFP is shown at the right. M, 100 bp size standards with selected sizes shown. WT, RNA from wild type minigene; -1G>T, RNA from mutant IVS4-1G>T minigene; -11A>G, RNA from mutant IVS5-11A>G minigene. B. Schematic of the 3'ss used in the IVS4-1G>T RT-PCR products from (A) that were cloned and sequenced. C. Schematic of the RNA sequence of patient IVS5-11A>G RT-PCR products from (A) that were cloned and sequenced. In B and C, exon sequences are boxed and in uppercase, intron sequences are in lowercase, and an arrow points to the patient mutation.

Patient 0175 reported here and patient 2 previously described by Lines et al. [25] display severe features of ARS (see above) and harbor a G>T mutation in the -1 position of the 3' ss of exon 4 mutation (IVS4-1G>T) (Figures 1A and 2C). This changes the canonical "AG" acceptor splice site to an "AT". In addition, a G>C substitution at the same site was previously reported in a patient that displayed classic ARS [18,25]. To test the effect of the G>T mutation on splicing, the three cell lines were transfected with WT or IVS4-1G>T minigenes and the splicing status of the mRNA was analyzed by RT-PCR. A single RT-PCR product of the appropriate size for correctly spliced RNA was observed with HEK293 (Figure 4A, top, lane 3), cornea stromal (bottom, lane 7), and HeLa cell RNA (not shown). A GFP-expressing plasmid used as a transfection and RNA loading control showed equal loading in each case (Figure 4A). To examine the possibility that a subtle change in splicing occurred that was not obvious from the size of the RT-PCR product, the PCR products were cloned and 13 HEK293 and three cornea cell independent clones were sequenced. All sequences showed that splicing was shifted 2 nt downstream to the next available "AG" dinucleotide (Figure 4B). Based on the results with the minigene in cultured cells, it is likely that all *PITX2 *mRNA from this allele is incorrectly spliced in these patients.

### A G>C mutation 5 nt downstream of the 5' splice site of exon 4 results predominantly in intron retention

**Figure 5:**
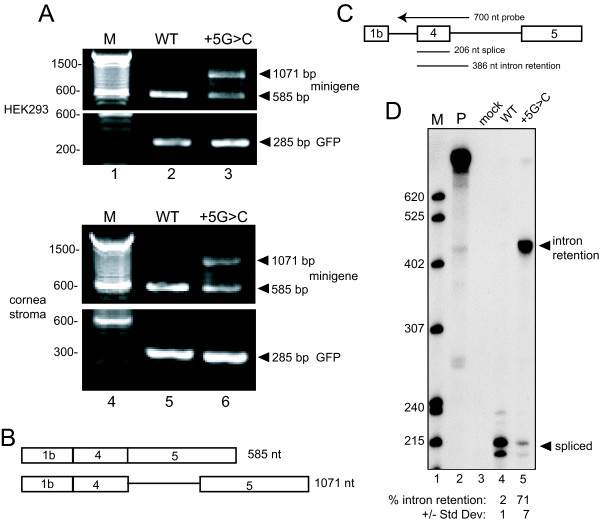
**The IVS4+5G>C mutation causes intron retention**. A. RNA from HEK293 cells (lanes 2 and 3) or human cornea stromal cells (lanes 5 and 6) transfected with WT or IVS4+5G>C minigenes and GFP was subjected to RT-PCR and products were resolved on an agarose gel. The positions of correctly spliced (585 bp), intron retention (1071 bp), and GFP products are shown at the right. M, 100 bp size standards with selected sizes shown. B. Diagram depicting the RNA structure of RT-PCR products from (A) that were cloned and sequenced. At the top is correctly spliced RNA, the bottom is RNA that retains the intron downstream of exon 4. C. Schematic of the RNase protection probe and protected products observed in D. The sizes of the probe and protected products are indicated. D. RNase protection assay (RPA). HEK293 RNA was subjected to RPA, protected products were resolved on a 6%–8 M urea polyacrylamide gel, and a phosphorimage is presented. Correct splicing generates a ~206 nt band and intron retention gives a ~386 nt band. M, ^32^P-labeled pBR322/MspI markers with sizes indicate on the left; P, undigested probe; mock, RPA using RNA from untransfected cells; WT, RNA from WT minigene-transfected cells; +5G>C, RNA from mutant IVS4+5G>C minigene-transfected cells. The positions of correctly spliced and intron retention protected products are shown at the right. The doublet bands for spliced product are likely due to incomplete RNase digestion. Below the lanes is quantitation of % intron retention (+/- standard deviation) as assessed by PhosphorImager analysis (n = 17).

The G>C mutation at the +5 position of the 5'ss of exon 4, IVS4+5G>C, was originally described in a family with ARS [9] and above we described the same mutation in a patient 689 with an isolated Axenfeld-Rieger anomaly. To assess the effect of this mutation on splicing, HeLa, HEK293, and human cornea stromal cells were transfected with wild type (WT) or mutant minigenes and GFP, and the isolated RNA was analyzed by RT-PCR. As shown in Figure 5A for HEK293 and cornea stromal cells, a band of the size expected for correctly spliced RNA was again observed from the WT minigene (lanes 2 and 5) but two RT-PCR products were seen with IVS4+5G>C (lanes 3 and 6). The lower band size was consistent with correctly spliced minigene RNA whereas the second product was larger but not the expected size for completely unspliced RNA. Analysis of GFP showed equal loading for each sample (Figure 5A). Similar results were seen with HeLa cell RNA (data not shown).

To determine the nature of the aberrant PCR product observed from the IVS4+5G>C minigene, both RT-PCR products from HEK293 RNA were cloned and independent clones of six large products and eleven 585 bp products were sequenced. The sequences showed that the lower band of IVS4+5G>C RNA was derived from correctly spliced mRNA and that the upper band retained the intron between exons 4 and 5 (Figure 5B).

To accurately quantitate the frequency of intron retention in IVS4+5G>C RNA, an RNase protection assay (RPA) was performed using a 700-nt riboprobe that spanned the 206 nt exon 4 and included upstream and downstream intron sequences. Correctly spliced RNA results in a ~206 nt protected band while retention of the intron would yield a 386 nt band (Figure 5C). RNA from the WT minigene was virtually completely spliced (Figure 5D, lane 4) whereas approximately 71% of the RNA from IVS4+5G>C retained the second intron (Figure 5D, lane 5). Thus, mutation IVS4+5G>C causes significant disruption in PITX2c minigene splicing.

### An A>G mutation 11 nt upstream of the 3' splice site of exon 5 shifts splicing to that site

The previously reported patients from family 4 [9] and family 5 [21] have classic ARS and harbor an A>G mutation 11 nt upstream of the 3' ss associated with *PITX2 *exon 5 (mutation IVS5-11A>G) (g.20745A>G as seen in *PITX2 *sequence GenBank # AF238048; Figure 2C). To determine if this mutation affects splicing, HeLa, HEK293 and human cornea stromal cells were transfected with the WT or IVS5-11A>G minigenes and the isolated RNA was analyzed by RT-PCR. The RT-PCR product from IVS5-11A>G RNA was the same size as correctly spliced *PITX2c *mRNA (Figure 4A, lanes 4 and 8). PCR of GFP served as a loading control. To determine if the mutation caused an aberrant splice that did not alter the RT-PCR product size appreciably, the RT-PCR product was cloned and 11 independent HEK293 clones were sequenced. All of the sequences showed that splicing was shifted exclusively to the newly created "AG" dinucleotide acceptor site 11 nt upstream of the authentic 3' ss (Figure 4C). Based on these results, it is likely that *PITX2 *mRNA from this allele is always incorrectly spliced in these patients.

### Analysis of PITX2c protein expression

**Figure 6:**
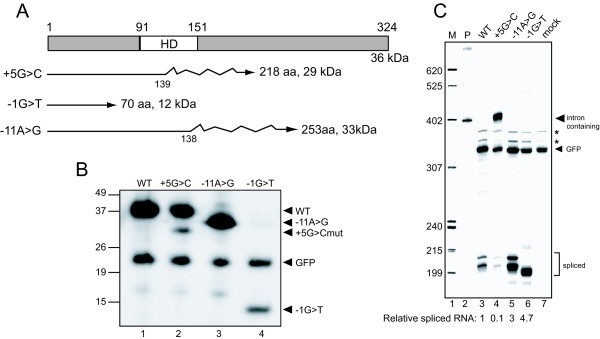
**Analysis of protein expression from mutant minigenes**. A. The domain structure of PITX2c protein with the position of the homeodomain (HD) is shown. Lines below represent the proteins that would result from aberrantly spliced RNA caused by each of the mutations. Straight lines indicate normal protein and jagged lines indicate incorrect amino acids generated by frame shifts; the number of normal amino acids and full-length size is shown for each. B. Western blotting of indicated FLAG epitope-tagged PITX2c and GFP proteins expressed in HEK293 cells. WT, protein from a wild type minigene transfection; +5G>C, protein from IVS4+5G>C mutant minigene transfected cells; -11A>G, protein from IVS5-11A>G mutant minigene transfection; -1G>T, protein from IVS5-11A>G mutant minigene transfected cells. The positions of wild type, +5G>C, -11A>G, and -1G>T PITX2 protein, and GFP protein, are indicated at the right. C. RNase protection assay (RPA). RNA from the indicated transfections was subjected to RPA with the same probe used in Figure 5, protected products were resolved on a 6%–8 M urea polyacrylamide gel, and a phosphorimage was obtained. Correct *PITX2c *splicing generates a ~206 nt band and GFP generates a 309 nt band. M, ^32^P-labeled pBR322/MspI markers with sizes indicated on the left; P, undigested probes; mock, RNA from GFP-transfected cells. The positions of correctly spliced and GFP protected products are shown at the right. The asterisks denote artifact bands derived from the GFP probe. The multiple bands for spliced product are likely due to incomplete RNase digestion. Below the lanes is quantitation of splicing relative to WT as assessed by PhosphorImager analysis.

Aberrantly spliced mRNA derived from the mutant patient *PITX2 *genes is expected to result in truncated PITX2c protein since frameshifts occur in each case (Figure 6A). PITX2 protein levels were determined in transfected cells by western blot (Figure 6B) after normalizing protein levels to GFP expression and RNA expression as determined by RPA. RPA analysis demonstrated that mRNA expression was roughly similar for each minigene (Figure 6C) and that, except for IVS4+5G>C, each minigene mRNA was doubly spliced (although aberrantly).

The WT minigene produced a protein of the expected size, ~36 kDa (Figure 6B, lane 1). The IVS4-1G>T minigene produced a much smaller protein (~12 kDa, lane 4), but this was expected since this protein would terminate at a single frame-shifted amino acid after PITX2 position 70. It appears that the IVS4-1G>T protein is unstable since it was present at only ~5% of WT levels, despite its mRNA being at least as abundant as WT. The IVS4+5G>C mRNA generated a normal sized protein and a second product of the size (~29 kDa) appropriate for truncated protein derived from intron-containing mRNA (lane 2); when corrected for mRNA levels, the truncated protein was about one tenth the abundance of normal protein. It was also evident that the level of normal protein expressed from the IVS4+5G>C minigene was roughly proportional to correctly spliced mRNA levels, which was about 30% of that produced from the WT minigene. Conversely, the level of truncated protein generated from the intron-retaining mRNA (~71% of minigene RNA produced) was considerably less than expected if protein expression was proportional to mRNA. These data are consistent with inefficient export of the intron-containing IVS4+5G>C mRNA from the nucleus. However, the possibility that the mRNA is exported but not translated, or exported but produces an unstable protein, cannot be excluded. Finally, the IVS5-11A>G mRNA would direct synthesis of a protein containing 138 residues of PITX2 followed by 117 frame-shifted amino acids and again, an expected ~33 kDa protein was expressed (lane 3) at a level similar to WT.

## Discussion

We report two new families with intronic mutations in the *PITX2 *gene and give insights into the mechanisms by which these and a previously described intronic mutation may affect PITX2 protein expression and influence development of ARS. A deficiency in normal PITX2 protein was proposed as the major cause of ARS and each of the mutations examined caused RNA splicing defects that would affect PITX2 protein levels or function, consistent with a gene dosage model for development of the syndrome.

Mutation IVS4-1G>T is the most 5' positioned *PITX2 *mutation identified to date ([18], [25], this report). This mutation changes the 3' intron-terminal G to a T and mutates the terminal "AG" intron junction dinucleotide. The terminal AG dinucleotide is critical for splicing catalysis and it was expected that splicing at the correct site would be abolished. A number of mechanisms for specifying the 3' ss AG have been proposed, including scanning from the branchpoint to the first AG [39], measurement from the branchpoint to the AG [40], or a combination of the AG being in the correct context and an optimal distance from the branchpoint [41]. The next available AG was located immediately 3' to the mutated junction and splicing was completely shifted to this site. Because of aberrant splicing, the resulting protein is predicted to contain only the N-terminal 70 amino acids of PITX2 and lack the entire homeodomain and C-terminal region. Extrapolation of our analysis in tissue culture cells to the patient suggests that the mutant protein would be present at negligible amounts and therefore, the patient's phenotype is most easily explained by haploinsufficiency of wild-type PITX2 protein (50% of normal). Interestingly, two unrelated patients with mutations at this nucleotide manifest ARS with one extra feature not commonly reported in ARS pedigrees – an anteriorly placed anus (this report; [18]). Also, two previously reported families with IVS4-1G>T and -1G>C substitutions were noted to display a severe form of Axenfeld-Rieger ocular anomaly including aniridia (unfortunately, detailed records were not available for patient 0175 reported here). Whether these features might be truly associated with this type of mutation and are caused by expression of only the N-terminal end of PITX2 remains to be seen.

The second mutation studied was identified in an individual (689) from a family with Axenfeld-Rieger anomaly and normal dental and umbilical development. The mutation is a change at position +5 of the 5'ss at the downstream border of exon 4 that led to predominant intron retention. This was surprising since exon skipping was the expected outcome. It is possible that our minigene/transfection system does not reflect the outcome in patients, but splice site mutations do result in intron retention at reasonable frequencies [42]. Curiously, the same mutation was previously reported in a family affected with complete ARS [9]. Little protein was generated from the aberrant mRNA and that which is made would be nonfunctional since it is truncated in the homeodomain. Since expression of the mutant allele in transfected cells resulted in 30% correctly spliced mRNA, the patient might be expected to have a milder phenotype since PITX2 protein would be ~65% of normal rather than the 50% expected of haploinsufficiency.

While our study demonstrates that the IVS4+5G>C mutation causes a similar level of intron retention in three tissue culture cell systems, it is curious that two individuals with the same mutation (families 689 and 2) exhibited different phenotypes and have corresponding clinical family histories (Figures 1B and 1C)[9]. Unfortunately, patient samples cannot be examined directly, but it is possible that the degree of aberrant splicing differs in these individuals, with a greater amount of aberrant splicing associated with a more severe phenotype. The mutation should alter recognition of the 5'ss by the core splicing machine, an event that is influenced by numerous positive and negative auxiliary factors whose expression patterns often show cell-type specific and temporal variation [32]. For example, trans-acting splicing activators often promote recognition of exons and correct splicing through interactions with splicing enhancer sequences located in exons. Indeed, purine-rich sequences upstream of the affected *PITX2 *5'ss resemble splicing enhancer elements that bind the SR protein family of splicing activators [43]. It is possible that genetic background differences influence the levels of one or more of these factors, resulting in differential effects of the IVS4+5G>C mutation on splicing in the two patients. The mutation would manifest as a less severe splicing defect, and thus higher levels of wild-type protein in the patient with the milder phenotype. It is also possible that the putative splicing enhancer elements in exon 4 explain the intron retention outcome by promoting recognition of the exon such that splicing of the upstream intron occurs, but the mutation in the 5' ss abolishes downstream intron splicing. Another possibility is genetic variation in the promoters of *PITX2 *target genes such that lower levels of PITX2 protein activate them.

The IVS4+5G>C *PITX2 *mutation represents another demonstration of affected phenotypes associated with a more than 50% level of normal PITX2 protein. In previous reports, it was argued that some of the PITX2 mutant proteins retain partial function and this fact explains milder ARS phenotypes seen in affected families [19,31]. Another observation emerging from these studies is that human ocular development appears to be the most sensitive to the amounts of PITX2 protein as "mild" mutations allow dental and umbilical development to proceed normally but still have a profound effect on ocular structures, while "severe" mutations result in more complex ocular defects (this report; [19,20,30,31]).

The third mutation (IVS5-11A>G) reported in two families with classic ARS is an A>G mutation located eleven nucleotides upstream of the 3' ss associated with exon 5 [9,21]. An important determinant of 3'ss strength is the polypyrimidine tract that lies between the branch point and the splice junction [32]; purine interruptions decrease 3'ss quality. The IVS5-11A>G mutation would not be expected to alter the strength of the 3' ss but the mutation does create a new "AG" dinucleotide and that could compete with the authentic one 11 nt downstream. As discussed above, this new AG could be recognized by scanning due to the fact that a pyrimidine tract is located upstream or by a mechanism similar to that described in Coffin-Lowry syndrome whereby the authentic AG in the *RSK2 *gene is involved in the first steps of splicing but the new AG is utilized in the second step as the splice junction [44]. Regardless, a complete shift in IVS5-11A>G minigene RNA splicing to the new site was observed and resulted in expression of a truncated protein that lacks a functional homeodomain. ARS phenotype in these families included classic ocular, dental and umbilical features (this report; Dr. Nishimura, personal communication) and can be explained by haploinsufficiency although the possibility that the mutant protein provides an additional effect cannot be excluded.

## Conclusion

In summary, we provide the first direct evidence that *PITX2 *intronic mutations cause splicing defects. We propose that the molecular defect in patients harboring these mutations is at the level of mRNA splicing such that the level of functional protein is reduced. Furthermore, the extent of the splicing defect observed in our system generally correlates with phenotypic severity, which supports the *PITX2 *gene dosage model for Axenfeld-Rieger syndrome. Because the mutations described here affect splicing of the last two exons that are common to all isoforms, similar splicing defects would be expected for all *PITX2 *transcripts. The gene dosage model was initially proposed based on in vitro studies that demonstrated an association between partial function for some mutant PITX2 proteins and milder phenotypes. Here, we report a splicing mutation (IVS4+5G>C) that results in production of some normal mRNA/protein from a minigene, which suggests an association between elevated levels of normal PITX2 (~65% vs. 50%) and milder human phenotypes. We also suggest that the phenotypic outcome may be dependent on secondary factors/genetic background of mutation carriers.

## Competing interests

The author(s) declare that they have no competing interests.

## Authors' contributions

NLM carried out the molecular studies in Figures 2 through 6 and participated in drafting the manuscript.

WLMA was involved in clinical evaluation of the ARS patients and provided human samples and clinical material for the study.

JCM provided collection of human patient and control samples and participated in genetic analyses.

EVS identified human mutations reported in the study, conceived and participated in the design of the study and helped draft the manuscript.

MTM conceived and participated in the design of the study, coordinated the molecular studies, helped draft the manuscript, and obtained study funding.

## Pre-publication history

The pre-publication history for this paper can be accessed here:


